# Transgenic and Mutation-Based Suppression of a *Berberine Bridge Enzyme-Like (BBL)* Gene Family Reduces Alkaloid Content in Field-Grown Tobacco

**DOI:** 10.1371/journal.pone.0117273

**Published:** 2015-02-17

**Authors:** Ramsey S. Lewis, Harry O. Lopez, Steve W. Bowen, Karen R. Andres, William T. Steede, Ralph E. Dewey

**Affiliations:** Department of Crop Science, North Carolina State University, Raleigh, North Carolina, United States of America; Louisiana State University, UNITED STATES

## Abstract

Motivation exists to develop tobacco cultivars with reduced nicotine content for the purpose of facilitating compliance with expected tobacco product regulations that could mandate the lowering of nicotine levels per se, or the reduction of carcinogenic alkaloid-derived tobacco specific nitrosamines (TSNAs). A *berberine bridge enzyme-like (BBL)* gene family was recently characterized for *N. tabacum* and found to catalyze one of the final steps in pyridine alkaloid synthesis for this species. Because this gene family acts downstream in the nicotine biosynthetic pathway, it may represent an attractive target for genetic strategies with the objective of reducing alkaloid content in field-grown tobacco. In this research, we produced transgenic doubled haploid lines of tobacco cultivar K326 carrying an RNAi construct designed to reduce expression of the *BBL* gene family. Field-grown transgenic lines carrying functional RNAi constructs exhibited average cured leaf nicotine levels of 0.684%, in comparison to 2.454% for the untransformed control. Since numerous barriers would need to be overcome to commercialize transgenic tobacco cultivars, we subsequently pursued a mutation breeding approach to identify EMS-induced mutations in the three most highly expressed isoforms of the *BBL* gene family. Field evaluation of individuals possessing different homozygous combinations of truncation mutations in *BBLa*, *BBLb*, and *BBLc* indicated that a range of alkaloid phenotypes could be produced, with the triple homozygous knockout genotype exhibiting greater than a 13-fold reduction in percent total alkaloids. The novel source of genetic variability described here may be useful in future tobacco breeding for varied alkaloid levels.

## Introduction

The pyridine alkaloids of tobacco (*Nicotiana tabacum* L.) are among the most studied group of plant secondary compounds in plants. Nicotine constitutes greater than 90% of the total alkaloid pool in most tobacco genotypes, and is primarily responsible for the pharmacological response experienced by users of tobacco products. In decreasing order of relative abundance, the remaining major alkaloids in tobacco include anatabine, nornicotine, and anabasine [1,2].

Alkaloid levels in tobacco are influenced by environmental conditions, interactions with plant pests, and plant genetics. The directed use of genetics to affect nicotine levels has been of interest to tobacco researchers since the 1930’s. For several reasons, motivation continues to exist today to investigate methods for reducing nicotine content in the tobacco plant. First, nicotine is the primary addictive substance found in tobacco products [3]. Under the Family Smoking Prevention and Tobacco Control Act of 2009, the United States Food and Drug Administration (FDA) was given the authority to regulate alkaloid levels in tobacco products, but cannot require the reduction of nicotine yields to zero. For the benefit of public health, some have studied the possible role of low-nicotine cigarettes in smoking cessation strategies [4,5]. Others have advocated for regulatory strategies where nicotine levels would be gradually reduced or immediately lowered to subaddictive thresholds [6,7]. Such proposals are not without controversy, however, as compensatory increased exposure to tobacco toxicants could occur with lower nicotine levels in tobacco products [5,8]. Even in the absence of directives on nicotine levels per se, this alkaloid will be an important component of future regulations because toxicant amounts will likely be reported per mg of nicotine in smoke [9].

Nicotine levels are also of interest to tobacco researchers because of the implicated role of nicotine as a precursor to one of the tobacco specific nitrosamines (TSNAs), a potent group of recognized carcinogens in tobacco products [10,11,12]. The most important TSNAs are *N*-nitrosonornicotine (NNN) and 4-(methylnitrosamino)-1-(3-pyridyl)-1-butanone (NNK), which are derived through nitrosation reactions with nornicotine and possibly pseudooxynicotine, respectively, during the curing, storage, and consumption of tobacco. Pseudooxynicotine is thought to be a degradation product of nicotine [13,14]. Reductions in NNN in cured leaves have been achieved through reduced expression of genes encoding for nicotine demethylase enzymes required for the conversion of nicotine of nornicotine, the precursor to NNN [15,16]. Likewise, reductions in NNK might be realized by lowering of nicotine and pseudooxynicotine levels. Because TSNAs are predominantly formed in air-cured tobaccos, one possible route for decreasing NNK in American blend cigarette products would be to utilize air-cured tobacco varieties with a reduced genetic potential to accumulate nicotine. Derived cured leaf might then be blended with appropriate flue-cured tobaccos with low TSNA levels to arrive at an overall low-TSNA tobacco product.

Large effects on tobacco alkaloid levels have historically been achieved through the use of naturally-occurring recessive alleles at the *Nic1* and *Nic2* loci (also designated as the *A* and *B* loci, in some literature). Recessive alleles at both of these loci can reduce alkaloid levels from between 1.5% and 4.5% to approximately 0.2% [17,18,19]. Although the *Nic1* locus remains uncharacterized, the *Nic2* locus was recently shown to encode for a cluster of ethylene response factor (ERF) genes [20]. Members of this cluster, exemplified by a gene designated as *ERF189*, appear to activate nicotine biosynthesis by binding to GCC-box elements found in the promoter regions of nicotine biosynthetic genes. Tobacco varieties carrying the recessive *nic1* or *nic2* alleles have not been widely used, however, because of negative associations with yield and/or quality [19,21,22].

**Fig 1:**
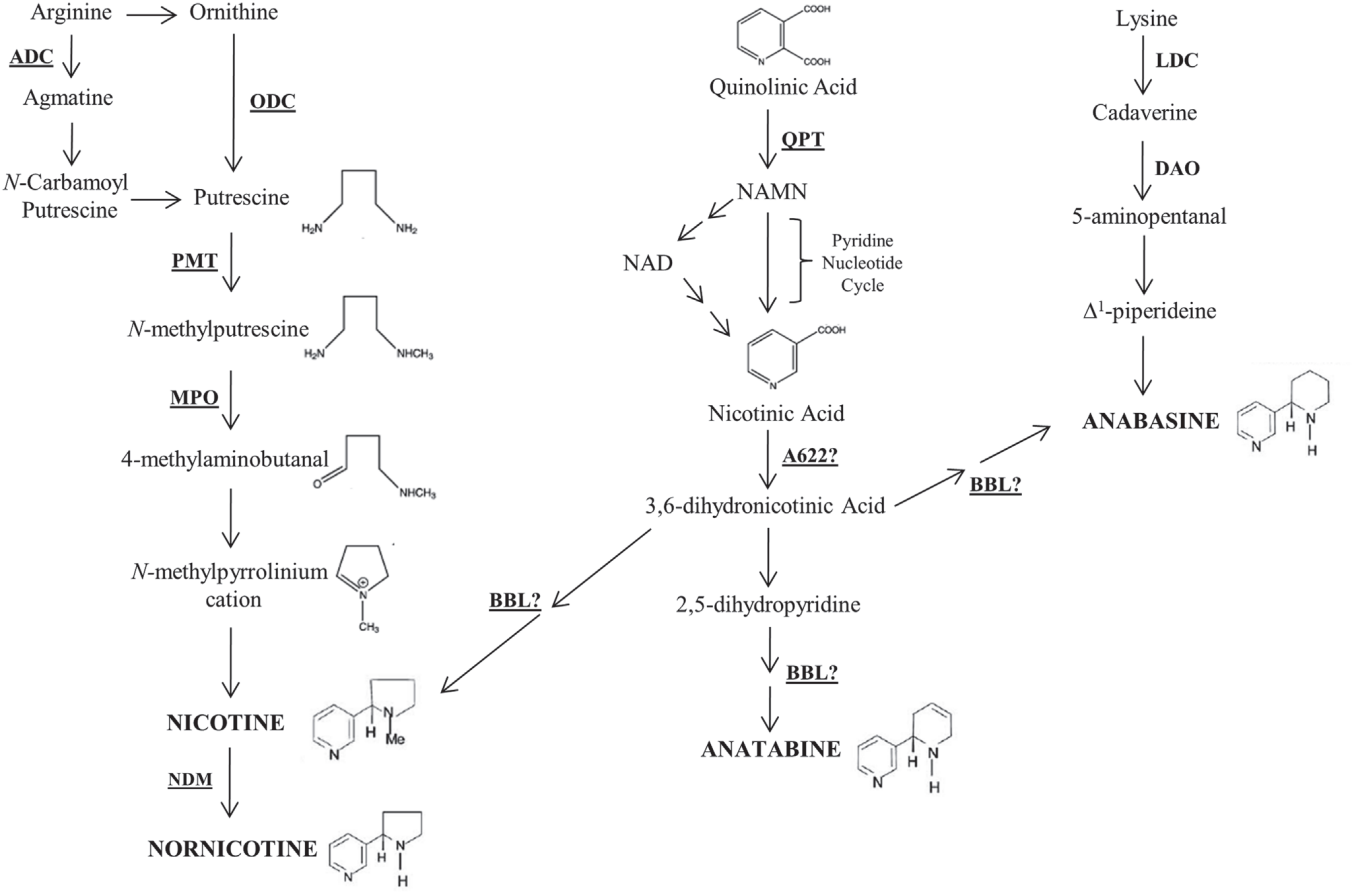
Model for alkaloid biosynthesis in *N*. *tabacum*. Known or suspected enzymatic steps are underlined: A622—isoflavone reductase-like protein; ADC—arginine decarboxlylase; BBL—berberine bridge enzyme-like; DAO—diamine oxidase; LDC—lysine decarboxlyase; MPO—*N*-methylputrescine oxidase; NDM—nicotine demethylase; ODC—ornithine decarboxlyase; PMT—putrescine methyltransferase; QPT—quinolinate phosphoribosyltransferase.

A great deal of knowledge has been gained in the last twenty years regarding the molecular biology underlying the biosynthesis of tobacco alkaloids [23]. Nicotine is comprised of pyrrolidine and pyridine rings that are each produced by an independent pathway of primary metabolism (Fig. 1). Synthesis takes place exclusively in the roots, where the corresponding biosynthetic enzymes can be found [13]. After synthesis, nicotine and related alkaloids are transported through the xylem to the leaves, where they accumulate within vacuoles [24]. Discovery of genes acting in the tobacco alkaloid biosynthetic pathway (Fig. 1) provides opportunities for genetically engineering the species for altered alkaloid content.

The *putrescine N-methyltransferase* (*PMT*) gene family encodes for the first committed step in the synthesis of the pyrrolidine ring of nicotine [25,26]. Transgenic *N*. *sylvestris* plants engineered to have reduced expression of the *PMT* gene family produced decreased nicotine content, but also exhibited leaf and inflorescence abnormalities [27]. These authors suggested that developmental problems may have been due to the accumulation of putrescine and polyamines, which may have roles in regulating plant development, and/or due to the accumulation of growth-altering alkaloid precursors from the pyridine branch of nicotine biosynthesis [27]. Reducing *PMT* expression in *Nicotiana* by co-suppression, antisense, or RNAi technologies also leads to dramatic increases in anatabine levels [28,29,30].

Genes encoding for *N*-methylputrescine oxidase (MPO) catalyze the second step in the biosynthesis of the pyrrolinium ring, which involves the oxidative deamination of *N*-methylputrescine to produce 4-methylaminobutanal (Fig. 1) [31,32]. Silencing of this gene family in tobacco hairy root cultures resulted in a phenotype similar to *PMT* suppression lines, where anatabine became the predominant alkaloid at the expense of nicotine [33].

The enzyme quinolinate phosphoribosyltransferase (QPT) catalyzes the entry point into the nicotinamide adenine dinucleotide (NAD) biosynthetic pathway in which nicotinic acid is an intermediate. Although some success has been achieved in lowering nicotine levels via QPT suppression [34], its usefulness as a target is likely to have limitations due to the essential role of enzyme step in primary metabolism.

The A622 enzyme (Fig. 1), a member of the PIP family of NADPH-dependent reductases, is predicted to be involved in the late stages of nicotine biosynthesis. Its role may include the production of a coupling-competent intermediate from nicotinic acid [35,36]. Initial efforts to generate whole transgenic tobacco plants with lowered *A622* expression levels have been unsuccessful, however, possibly due to a build-up of nicotinic acid which can be detrimental to cell survival and growth at high levels [35].

A gene family designated as *berberine bridge enzyme-like* (*BBL)* has only recently been found to play a role in tobacco alkaloid formation. The BBL enzyme is a flavin-containing oxidase predicted to be involved in the final oxidation step for nicotine production [37]. The impact of suppressing expression of the *BBL* gene family in multiple laboratory and greenhouse systems led to reduced nicotine phenotypes [37]. These authors also reported the accumulation of a novel metabolite identified as dihydrometanicotine (DMN) in the roots of transgenic *BBL*-suppressed tobacco plants, but not in leaf tissue, possibly due to the inability of cellular transporters to mobilize this novel alkaloid. Importantly, suppression of BBL activity in tobacco was not accompanied by any apparent defects in plant growth and development, nor was its reduction correlated with significant increases of other pyridine alkaloids.

The observations of Kajikawa et al. [37] suggest that the *BBL* gene family may be an ideal target for researchers with the objective of lowering nicotine content in tobacco cultivars. The objectives of this research were to (1) produce doubled haploid (DH) RNAi tobacco lines silenced for *BBL* expression, (2) evaluate the effect of *BBL* suppression on alkaloid levels, yield, and cured leaf quality in field-grown tobacco plants, and (3) determine the relative roles of three different *BBL* isoforms on alkaloid accumulation by evaluating field-grown tobacco plants carrying different combinations of induced mutations in these three genes. The information and derived genetic materials reported here may be useful to tobacco breeders desiring large decreases in nicotine content using a genetic engineering approach, or those desiring incremental decreases in nicotine content modulated by combining deleterious mutations in *BBL* genes.

## Results

### Silencing of the *BBL* Gene Family Through RNAi Suppression

To establish whether silencing the *BBL* gene family represents a viable means of obtaining reduced nicotine tobacco plants under conventional field growth conditions, we employed an RNAi approach (S1 Fig.). Kajikawa et al. [37] reported four unique *BBL* isoforms, designated *BBLa*, *BBLb*, *BBLc* and *BBLd*. To maximize the probability of suppressing the entire gene family, a 212 bp fragment from the most highly conserved region was selected. The anti-*BBL* RNAi construct was generated specifically against the *BBLa* sequence, as an in silico analysis of the tobacco EST sequences represented in GenBank suggested that it was the most highly expressed of the *BBL* isoforms (Table S1). The 212 bp fragment from *BBLa* is 94%, 93% and 84% identical to the analogous regions of *BBLb*, *BBLc* and *BBLd*, respectively. Although anti-*BBL* RNAi construct shares the least sequence identify with *BBLd*, both the in silico EST analysis (Table S1) and a reverse transcriptase PCR analysis conducted by Kajikawa et al. [37] suggest that the *BBLd* isoform is minimally expressed within the plant.

Flue-cured tobacco cultivar ‘K326’ was transformed with the anti-*BBL* RNAi construct and ten independent *35S*:*BBL*-RNAi DH lines were selected for evaluation in replicated field experiments for alkaloid profiles, yield, and cured leaf quality. Six of the ten tested RNAi lines exhibited nicotine levels in cured leaf that were significantly lower (*P* < 0.05) than that observed for the untransformed control line, K326 (Table 1). The remaining four RNAi lines produced nicotine levels that were roughly equivalent to that of K326. This is likely because the RNAi mechanism was not functioning in these transgenic lines. We attempted both RNA blot and PCR-based expression analyses from the roots of the field-grown plants to investigate the relationship between nicotine accumulation and *BBL* transcript suppression, but excessive degradation of the RNA preparations and/or residual soil contaminants prevented us from obtaining meaningful data. Furthermore, transcript analysis on leaf tissue would not be informative, given that *BBL* gene expression is highly root specific [37], Table S1). Transgenic line DH32 exhibited the lowest percent nicotine in cured leaf (0.414%), while untransformed K326 produced 2.454% nicotine. The lowest nicotine level (0.299%) was produced by LAFC53, a *nic1/nic1 nic2/nic2* isoline of flue-cured tobacco cultivar NC95.

**Table 1:** Means for measured characteristics for transgenic RNAi doubled haploid lines and associated checks.

	Root						Cured Leaf										
Genotype^a^	% Nico-Tine	% Nornicotine	% Anabasine	% Anatabine	% DMN	% Total Alkaloids	% Nicotine	% Nornicotine	% Anabasine	% Anatabine	% DMN	% Total Alkaloids^b^	% Reducing Sugars	Yield (kg ha^-1^)	Cwt Value ($ cwt^-1^)	Cash Return ($ ha^-1^)	Grade Index
K326 BBL-RNAi DH25A	0.4905	0.0239	0.0162	0.1011	0.0276	0.6592	2.4695	0.0542	0.0236	0.1205	0.0081	2.6758	12.97	2253	291.40	7223.03	77.9
K326 BBL-RNAi DH35	0.7072	0.0282	0.0186	0.0726	0.0121	0.8387	2.6376	0.0521	0.0222	0.1462	0.0096	2.8677	13.66	2128	318.63	7103.49	84.2
K326 BBL-RNAi DH43	0.5789	0.0261	0.0186	0.0611	0.0112	0.6959	2.6592	0.0532	0.0215	0.1297	0.0100	2.8735	13.73	2141	309.33	7522.17	82.3
K326 BBL-RNAi DH25C	0.5847	0.0256	0.0192	0.0490	0.0240	0.7025	2.4793	0.0450	0.0222	0.1152	0.0103	2.6720	15.04	2158	307.75	7342.08	81.5
K326 BBL-RNAi DH22A	0.1516	0.0354	0.0072	0.0176	0.0785	0.2903	0.5362	0.0784	0.0124	0.0143	0.0152	0.6565	13.71	1685	315.43	6439.64	83.3
K326 BBL-RNAi DH32	0.0550	0.0615	0.0054	0.0048	0.1522	0.2790	0.4136	0.0890	0.0132	0.0073	0.0209	0.5440	12.67	1628	316.78	6551.81	83.7
K326 BBL-RNAi DH16A	0.0472	0.0367	0.0037	0.0051	0.0797	0.1723	0.4720	0.0789	0.0125	0.0079	0.0163	0.5876	13.79	1439	309.34	4774.54	80.1
K326 BBL-RNAi DH19	0.0751	0.0480	0.0053	0.0079	0.1256	0.2620	1.0442	0.0864	0.0178	0.0358	0.0165	1.2007	12.91	1615	322.99	5800.20	85.5
K326 BBL-RNAi DH303	0.1456	0.0662	0.0091	0.0156	0.1740	0.4106	0.9110	0.0859	0.0166	0.0233	0.0178	1.0547	14.08	1944	311.13	7166.99	83.3
K326 BBL-RNAi DH16B	0.0491	0.0421	0.0039	0.0055	0.0920	0.1926	0.7288	0.0781	0.0147	0.0283	0.0159	0.8658	13.32	1591	317.49	5831.29	84.0
K326	0.7008	0.0303	0.0259	0.0739	0.0112	0.8420	2.4541	0.0559	0.0206	0.1188	0.0104	2.6598	14.71	2343	317.29	8254.99	83.9
NC95	0.4149	0.0466	0.0157	0.0587	0.0725	0.6084	2.8870	0.0636	0.0303	0.2217	0.0099	3.2125	12.95	1722	306.66	6105.61	81.3
LAFC53	0.3922	0.0101	0.0113	0.0603	ND^c^	0.4739	0.2990	0.0128	0.0035	0.0201	0.0074	0.3428	8.55	1844	303.98	6519.67	79.4
LSD (0.05)	0.1748	0.0279	0.0061	0.0393	0.0724	0.1655	0.3998	0.0160	0.0032	0.0216	0.0039	0.4067	3.59	304	25.64	1098.66	6.4

^a^ For convenience, genotypes are presented in three groups: four RNAi lines with non-significant changes in nicotine content, six RNAi lines with significant observed reductions in nicotine content, and three non-transgenic checks.

^b^ % total alkaloids was calculated as: % total alkaloids = (% nicotine + % nornicotine + % anatabine + % anabasine + % DMN).

^c^ ND = non-detectable

All six of the RNAi lines with significantly lower percent nicotine relative to K326 also exhibited significantly (*P* < 0.05) lower anatabine and total alkaloid levels (Table 1) in the cured leaf. Five of these six lines also exhibited significantly (*P* < 0.05) lower levels of anabasine. All six were significantly higher for percent nornicotine and percent DMN, although these numerical differences were very small.

Alkaloid profiles were also determined for root samples collected for each plot at one field location after the completion of leaf harvests. Results roughly mirrored those for the cured leaf samples. All six RNAi lines with significantly reduced nicotine levels in the cured leaves also exhibited significantly reduced (*P* < 0.05) nicotine, anatabine, anabasine, and total alkaloid levels in root tissues relative to samples from non-transformed K326 (Table 1). Two RNAi lines had significantly greater accumulation (*P* < 0.05) of nornicotine in their roots relative to K326, and four lines exhibited significantly higher levels of DMN. DMN became the most prevalent root alkaloid in five of the transgenic lines.

The six RNAi lines exhibiting significantly lower nicotine and total alkaloid levels relative to K326 produced significantly lower (*P* < 0.05) cured leaf yields as compared to untransformed K326, with an average difference of 693 kg ha^-1^ (Table 1). Such large yield differences were not observed for the four transgenic DH lines where the RNAi mechanism was apparently not functioning. No significant differences were detected between K326 and any of the RNAi lines for cured leaf quality as measured by percent reducing sugars, value per hundredweight ($ cwt^-1^), or grade index.

### Identification and Testing of Induced *BBL* Gene Mutations

A second major objective of this research was to identify deleterious mutations in *BBLa*, *BBLb*, and *BBLc* in an EMS mutant population in order to determine the relative effects of each of these isoforms on alkaloid accumulation and to also permit for development of a non-transgenic tobacco genotypic series with the potential to produce a range of alkaloid phenotypes. We did not screen for mutations in *BBLd* because, as described above, this isoform appears to be minimally expressed in comparison to the other three. Of 1,248 plants screened for *BBLa*, nine missense mutations and one truncation mutation were identified (Table 2). For *BBLb*, six missense mutations and one truncation mutation were observed across 1,344 plants surveyed (Table 3). Of 2,112 plants screened for mutations in *BBLc*, eleven plants were found to possess missense mutations, and a single plant was identified as a truncation mutant (Table 4).

**Table 2:** Position and effect of mutations identified in the *BBLa* gene in the mutated genetic background of tobacco line DH98–325–6.

Mutant	Mutation	Position from ATG	Amino Acid Change	SIFT Prediction
62	G/A	450	R150K	Tolerated
507	G/A	267	R89H	Tolerated
2610	G/A	978	E326K	Damaging^a^
2327	G/A	1194	E398K	Damaging
2665	C/T	1263	A421V	Tolerated
2977	G/A	351	V117M	Damaging
3118	G/A	663	G221S	Tolerated
1705	G/A	375	D125K	Tolerated
1265	G/A	255	R85K	Tolerated
1435	G/A	681	W227Stop	N/A

Total # of plants screened: 1,248

# missense mutations identified: 9

# truncation mutation mutations identified: 1

silent mutations not included

^a^ Mutations predicted to be damaging had a SIFT score < 0.05.

**Table 3:** Position and effect of mutations identified in the *BBLb* gene in the mutated genetic background of tobacco line DH98-325-6.

Mutant	Mutation	Position from ATG	Amino Acid Change	SIFT Prediction
875	G/A	554	G185D	Damaging^a^
1347	C/T	578	L190F	Tolerated
2890	G/A	655	A219N	Tolerated
2704	C/T	589	L197F	Damaging
941	C/T	370	P124S	Tolerated
3061	G/A	529	V177I	Tolerated
675	G/A	438	W146Stop	N/A

Total # of plants screened: 1,344

# missense mutations identified: 6

# truncation mutation mutations identified: 1

silent mutations not included

^a^ Mutations predicted to be damaging had a SIFT score < 0.05.

**Table 4:** Position and effect of mutations identified in the *BBLc* gene in the mutated genetic background of tobacco line DH98-325-6.

Mutant	Mutation	Position from ATG	Amino Acid Change	SIFT Prediction
231	G/A	496	A166T	Damaging^a^
86	G/A	524	G175E	Damaging
1263	G/A	664	A222T	Damaging
1763	C/T	746	T249I	Damaging
948	C/T	920	P307L	Damaging
1606	G/A	544	G182S	Damaging
1570	G/A	472	A158T	Tolerated
1080	G/A	325	A109R	Damaging
604	C/T	604	L202F	Damaging
478	G/A	563	R188K	Damaging
1107	G/A	527	G176S	Damaging
1771	C/T	448	Q150Stop	N/A

Total # of plants screened: 2,112

# missense mutations identified: 11

# truncation mutation mutations identified: 1

silent mutations not included

^a^ Mutations predicted to be damaging had a SIFT score < 0.05.

**Fig 2:**
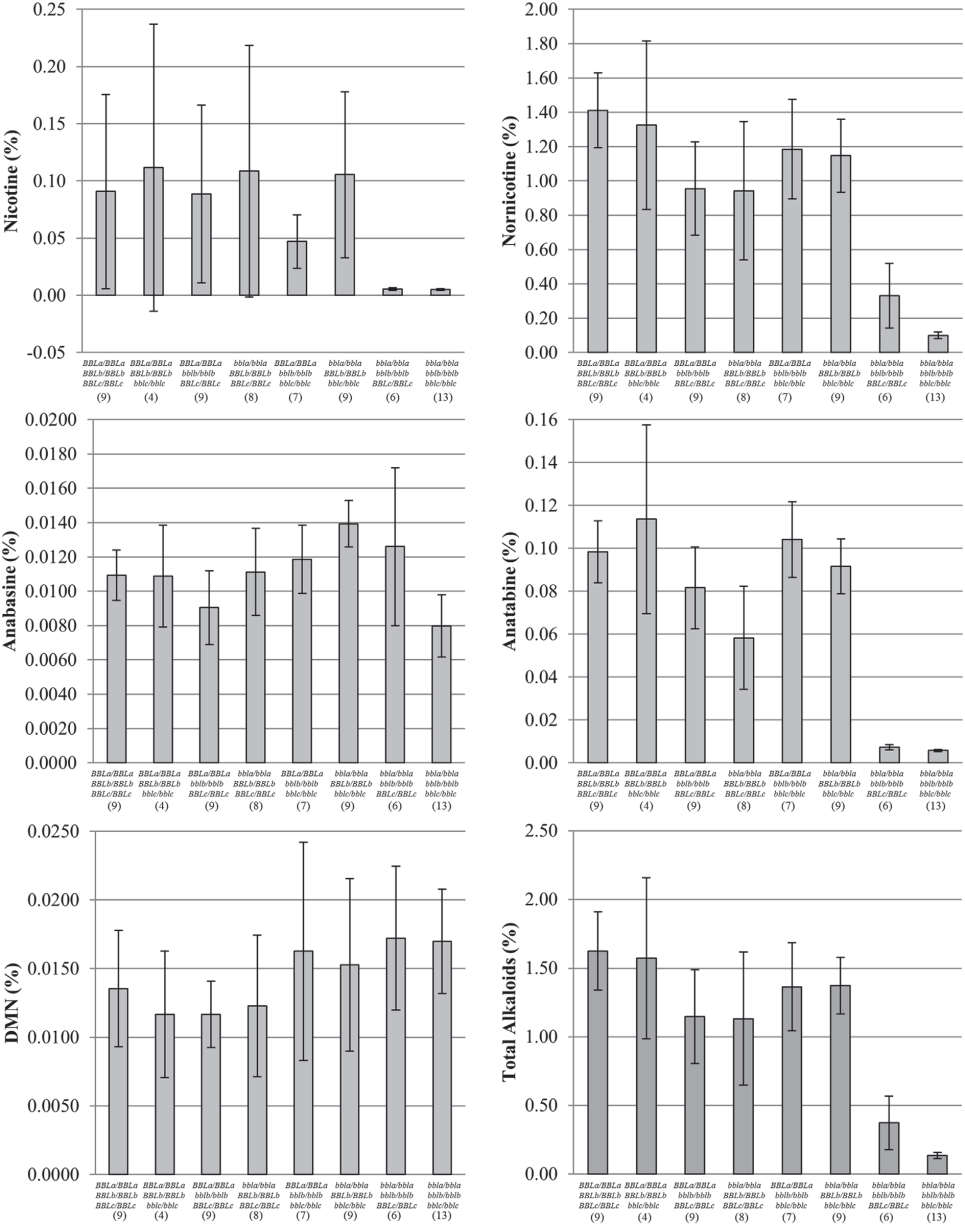
Alkaloid means for eight *BBL* knockout mutant combinations in a DH98–325–6 genetic background. Numbers of plants characterized for each genotypic class are shown in parenthesis. Confidence intervals (95%) are provided.

Sexual crossing combined with SNP genotyping were used to combine identified truncation mutations in *BBLa*, *BBLb*, and *BBLc* in all possible homozygous combinations in the mutagenized DH98–325–6 genetic background. Plants of all seven possible genotypic classes were compared for alkaloid profiles to wild-type segregants grown in a single field environment (Fig. 2). Wide numerical ranges were observed among the genotypic classes for percent nicotine, nornicotine, anatabine, and total alkaloids. Nornicotine was the most prevalent alkaloid in all of these genotypes because DH98–325–6 has a high genetic potential to convert nicotine to nornicotine due to an active nicotine demethylase gene designated as *CYP82E4* [16]. Genotypes homozygous for single mutations exhibited slight to intermediate reductions in total alkaloids. Of the three single mutation genotypes, the *bbla*/*bbla* mutation was found to have the largest numerical effect, while the *bblb*/*bblb* mutation was found to have the second largest effect. The *bblc*/*bblc* mutation, by itself, provided only a small reduction in percent total alkaloids. The double mutant combination *bbla*/*bbla bblb*/*bblb BBLc*/*BBLc* and the triple homozygous mutation combination *bbla*/*bbla bblb*/*bblb bblc*/*bblc* exhibited the second lowest and lowest levels of total alkaloid accumulation, respectively. These levels were substantially and significantly lower than that for wild-type DH98–325–6 segregants (Fig. 2). The results for percent nornicotine and anatabine mirrored very closely those for percent total alkaloids. Percent DMN was highest for the *bbla*/*bbla bblb*/*bblb BBLc*/*BBLc* and *bbla*/*bbla bblb*/*bblb bblc*/*bblc* mutant combinations, although their levels were not significantly different from that of wild-type DH98–325–6.

## Discussion

Prior to commercialization, tobacco varieties currently grown in the United States must exhibit nicotine and total alkaloid levels within specific windows defined with reference to certain long-term check varieties [38,39]. Nevertheless, there is motivation to consider development of tobacco varieties with reduced alkaloid content. Some members of the health community advocate for reductions in nicotine yields of smoking products as part of tobacco use control strategies [5,6,7]. Reduced alkaloid tobacco varieties might also be of value in overall strategies to reduce smokers’ exposure to carcinogenic alkaloid-derived TSNAs. Genetic approaches to reduce nicotine synthesis and total alkaloid accumulation in tobacco include: (1) use of naturally existing allelic variability, including the large effect recessive alleles *nic1* and *nic2* [17,18], (2) transgene-mediated suppression of genes involved in the alkaloid biosynthetic pathway, and (3) induced mutagenesis of genes necessary for alkaloid production. Extensive research has been reported on the use of the recessive *nic1* and *nic2* alleles to reduce alkaloid levels in cured tobacco, but use of this variability is associated with negative effects on yield and/or cured leaf quality [19,21,22,29]. The *Nic2* locus is known to encode for a series of transcription factors [20] that globally influence the expression of a suite of genes involved in alkaloid biosynthesis [20,25,40,41], and possibly other genes that may affect the physiology, stress response, and senescence of the tobacco plant [19,42].

The *BBL* gene family was previously reported to act in the final stages of nicotine synthesis [37]. Lowering expression of genes in this family might be expected to have fewer undesired effects on traits outside of alkaloid accumulation as compared to the unfavorable side effects caused by reduced expression of the global regulatory factors encoded by *Nic1* and *Nic2*. Consistent with the results of [37], we identified four *BBL* isoforms with variable levels of expression. Evaluation of transgenic tobacco doubled haploid lines carrying an RNAi construct designed to down-regulate expression of the *BBL* family indicated that reduced expression can have a dramatic effect to reduce nicotine, anatabine, anabasine, and total alkaloid levels in cured leaf produced by field-grown tobacco plants. The percent nicotine level for the lowest nicotine accumulating RNAi line was 0.414%, while the untransformed check produced 2.454% nicotine. This reduction was not numerically as low as that provided by the *nic1/nic1 nic2/nic2* LAFC53 genotype, however (0.299% nicotine).

Cured leaf yields were found to be significantly lower for many of the transgenic RNAi lines (Table 1). This could be partially attributed to deleterious somaclonal variation introduced during the tissue culture-based transformation and chromosome doubling procedures [43]. The fact that such large reductions were not observed in the transgenic lines where the RNAi mechanism was apparently not functioning, however, suggests otherwise. Further research needs to be conducted to determine possible explanations for this effect. Abnormal accumulation of upstream metabolites such as certain polyamines could possibly adversely affect plant development and dry weight accumulation. Cured leaf quality for the transgenic materials was found to be equivalent to that produced by the non-transformed check variety. This would be advantageous, as the *nic1* and *nic2* system for reducing alkaloid content has been reported to be associated with reduced cured leaf quality [19,21,22].

Genetic engineering to alter expression of the *BBL* gene family has a number of advantages over transgenic manipulation of the expression of alternative alkaloid biosynthetic genes. Prior transgenic studies have shown that suppression of *PMT* genes via antisense or RNAi technologies leads to an accumulation of putrescine (PMT’s substrate) and dramatic increases in levels of anatabine, an alkaloid generated solely through the pyridine pathway [28,29,30]. It was speculated that the increased anatabine levels may be due to a detoxification mechanism that helps maintain nicotinic acid at a low level (due to its toxic effect on plant cells) at times when there may be disruption in the pyrrolinium synthesis pathway [28]. Blocking the enzymatic steps catalyzed by MPO and ODC resulted in similar changes in alkaloid profiles [33,44]. Although targeting *PMT* has been shown to be effective in lowering nicotine levels, the simultaneous increase in anatabine to levels far beyond that naturally found could deter the acceptance of tobacco varieties produced in this manner.

Other genes that have been targeted as a means to reduce nicotine levels are *QPT* and *A622*. The QPT enzyme was initially targeted because of its key role in regulating the synthesis of nicotinic acid [34]. Lowering *QPT* expression levels may not be an efficient strategy, however, due to possible undesirable consequences with respect to primary metabolism related to the pyridine nucleotide cycle. Lowering levels of the A622 enzymes may also be impractical because of accumulation of a possibly toxic nicotinic acid-derivative that may be detrimental to cell survival and plant growth [35].

In addition to the physiological reasoning outlined above, the transgenic *BBL*-based approach to reducing alkaloid levels offers additional advantages: (1) single RNAi constructs can reduce the expression of multiple, closely related genes, and (2) the RNAi-induced trait is consequently controlled by a single dominant gene, a feature that can greatly reduce the complexity of subsequent breeding of the trait. The efficiency of transgenic technologies is often variable, however, and the conferred trait can become unstable after a number of generations [45]. Moreover, the commercial deployment of transgenic tobacco is complicated by the various obstacles that must be overcome in order to deploy transgenic commercial crops. Major concerns and issues include: (1) the potential rejection of derived products by consumers, (2) the need to obtain costly licenses for enabling technologies required for the production of transgenic plants, and (3) the potentially long and costly process of deregulating transgenic traits.

The chemical mutagenesis strategy offers several advantages over transgenic modifications, both for functional studies and practical agricultural application [46]. This non-transgenic approach has the potential of generating an allelic series for any given locus, including missense mutant alleles and truncation mutations. This would make a range of alkaloid phenotypes possible, with an accumulation of all possible knockout mutations providing the opportunity for exceptionally low alkaloid levels. Mutations generated in this manner are stable and can be easily introduced into breeding varieties for commercial use. Derived lines could provide an alternative source of low alkaloid tobacco plants that circumvent hurdles associated with the commercialization of transgenic plants.

In this paper, we report the successful identification of truncation mutations in the three most highly expressed *BBL* genes. As expected, different genotypic combinations of the induced mutations provide a range of alkaloid phenotypes, with the triple homozygous mutant genotype providing the lowest alkaloid levels. Percent total alkaloid levels were reduced from 1.612% for wild-type genotypes to 0.119% for triple homozygous mutant genotypes, an approximate 93% reduction. Comparison of homozygous single mutation genotypes indicated that the *BBLa* isoform has the largest effect on alkaloid phenotypes, and revealed *BBLc* to have the smallest effect. A strict comparison of the mutation results with the RNAi results cannot be made for leaf chemistry because the genetic technologies were evaluated in different genetic backgrounds, with the mutation-based approach being evaluated in a strong nicotine-converting background. A comparison amongst the mutation-derived materials for yield and cured leaf quality was not carried out because the heavy mutation load in the current materials would have made an appropriate comparison difficult. We are currently in the process of transferring the three deleterious *BBL* mutations to a K326 background, which will permit appropriate direct comparisons. This should allow for a determination of the relative effect of the minimally expressed *BBLd* isoform. Although this isoform is expressed at low levels, it is possible that the corresponding gene product could still play a significant role in nicotine synthesis. Furthermore, with the recent publication of a reference genome for tobacco, it appears that there may be additional “minor” *BBL* genes present in the genome that could contribute toward alkaloid biosynthesis [47].

Suppressing expression of the *BBL* gene family is an effective tool to reduce nicotine and total alkaloid content of cured leaves of field-grown plants. It should be noted, however, that levels of alternative alkaloids can also be affected. First, the levels of anatabine and anabasine are concurrently reduced along with nicotine levels in field-grown tobacco plants. These observations are consistent with the greenhouse and laboratory observations of Kajikawa et al. [37]. Such results led Kajikawa et al. [37] to suggest that, because the biosynthesis of nicotine, anatabine, and anabasine all involve late-stage condensation reactions involving a pyridine ring, *BBL* gene products are likely involved in the activation of nicotinic acid or condensation reactions yielding bicyclic pyridine alkaloids [37].

DMN, a minor alkaloid whose presence in tobacco has been largely overlooked or ignored to date, became the most prevalent alkaloid in the roots of many of the transgenic RNAi lines. This alkaloid is apparently transferred to above-ground plant parts with very low efficiency, however, as nicotine remained the most abundant alkaloid in cured leaves. Although DMN levels were found to be significantly increased in the cured leaves of many RNAi lines, their absolute levels remained very low. This is in contrast to the results of Kajikawa et al. [37] who were not able to detect this novel metabolite in aerial plant parts. Nornicotine levels (at least with respect to its ratio with nicotine) may also be slightly increased in *BBL*-suppressed plants. Nornicotine is usually a minor component of the alkaloid fraction in the leaves and roots of *N*. *tabacum*, and most of its synthesis occurs directly by the enzymatic *N*-demethylation of nicotine during leaf senescence [15,48]. Recent studies have suggested, however, that a minor amount of nornicotine may be produced by an alternative pathway that does not involve nicotine *N*-demethylase (NDM) enzymes, or nicotine as an intermediate [16]. It would be interesting to investigate the phenotype of an RNAi-*BBL* construct expressed within a tobacco background deficient in NDM activity. Should nornicotine levels rise significantly in these plants, this would provide compelling data in support of an alternative nornicotine biosynthetic pathway.

## Materials and Methods

### Generation and Evaluation of Transgenic Plant Materials

Flue-cured tobacco cultivar ‘K 326’ was transformed with an RNAi construct engineered to down-regulate expression of the *BBL* gene family. The RNAi-based antisense construct directed against the *BBL* gene family was constructed in the vector pKYLX71 in a manner similar to that described by [48]. A 212 bp segment of the *BBLa* isoform that is highly conserved among all four identified *BBL* isoforms was amplified (positions 245–457 from the start ATG codon). To facilitate subsequent cloning steps, primers were designed to add a *Hind*III and *Xho*I site on the ends of the sense arm (5′-GCAAAGCTTGCACCATTTTTTGTTGCAGAAAAGC-3′ and 3′-GGCCTCGAGGGCAATGGCATAATAAATTTGGC-5′) and a *XbaI* and *SacI* site on the ends of the antisense arm (5′-GCATCTAGAGCACCATTTTTTGTTGCAGAAAAGC-3′ and 3′-GCCGAGCTCGGCAATGGCATAATAAATTTGGC-5′). Each fragment was cloned into the pCR2.1 vector (Invitrogen, Carlsbad, CA) and subsequently digested with *Xba*I and *Sac*I for the antisense insertion, and with *Hind*III and *Xho*I for the sense insertion (S1 Fig.). The *BBLa* sense fragment was subcloned into pKYLX80, followed by insertion of the antisense fragment. This intermediate vector enables the cloning of the sense and anti-sense fragments separated by a 151 bp soybean ω-3 fatty acid desaturase intron to enhance stability of the construct in bacteria as well as increasing the efficiency of silencing in the plant. The *BBLa* cassette from pKYLX80 was then digested with *Hind*III and *Sac*I and ligated into the plant expression vector pKYLX71 to produce the final anti-*BBL* RNAi construct. The pKYLX71 RNAi construct contains the *Cauliflower mosaic virus* (*CaMV*) 35S promoter that drives expression of the anti-*BBLa* cassette and *neomycin phosphotransferase II* (*npt*II) gene to allow for selection of transformed plants in the presence of kanamycin (S1 Fig.).

The pKYLX71 RNAi construct was introduced into *Agrobacterium tumefaciens* strain EHA105. A doubled haploid (DH) approach was used to produce transgenic tobacco lines carrying the RNAi construct. Haploid K326 leaf tissue was produced using the method of Burk et al. [49] and transformed with the pKYLX71 *BBL* RNAi construct according to the procedure of An et al. [50]. Regenerated transgenic haploids were chromosome doubled using the midvein culture method of Kasperbauer and Collins [51] to produce DH lines homozygous for the RNAi transgene insertion(s). Single primary transformants (R_0_ generation) were self-pollinated in a greenhouse to produce R_1_ seed.

### Developing and Screening Tobacco EMS Mutant Populations

A previously described EMS-induced mutation population in the background of burley tobacco breeding line DH98–325–6 [16] was used to identify mutations in the *BBLa*, *BBLb*, and *BBLc* isoforms. Briefly, seeds were mutagenized by soaking in 0.5% EMS for 12 hrs, washed with dH2O (8X) and then sown. The resulting M1 population was allowed to self-pollinate and leaf tissue from individual M2 plants was used for preparation of genomic DNA samples. PCR primers were designed for specific amplification of each targeted *BBL* isoform (Table S2). PCR reactions contained 1 μl of genomic DNA (~40 ng), 10 pmoles of each primer, 0.2 mM dNTPs (Roche Applied Science, Mannheim, Germany) and 1.4 U of Taq DNA polymerase (New England Biolabs, Beverly, MA) in a total volume of 15 μl. PCR conditions were 94°C for 3 min, followed by 35 cycles at 94°C for 35 sec, 54°C (*BBLc*) or 57°C (*BBLa* and *BBLb*) for 30 sec, and 72°C for 1 min, and a final extension at 72°C for 7 min. Fidelity of amplification products was confirmed by electrophoresis on 1% agarose gels.

PCR products were cleaned using the Shrimp Alkaline Phosphatase (1U/μl; Roche Applied Science, Mannheim, Germany) and Exonuclease I (20U/μl; New England Biolabs) protocols to prepare samples for DNA sequencing (http://www.nucleics.com/DNA_sequencing_support/exonucleaseI-SAP-PCR-protocol.html). PCR amplification products were directly sequenced using either nested primers or one of the original PCR primers in a cycle sequencing labeling reaction with Big Dye Terminators (Applied Biosystems, Foster City, CA). Sequences were analyzed on an ABI 3730 DNA analyzer (Applied Biosystems, Foster City, CA) and later aligned using the AlignX tool of Vector NTI (Invitrogen, Carlsbad, CA). All sequences obtained were screened for single base pair changes in the coding regions of the corresponding *BBL* genes.

### Field Evaluation of Transgenic *BBL*-RNAi Materials

A total of ten independent *35S*:*BBL*-RNAi DH R_1_ lines were selected for replicated field evaluation for alkaloid profiles, yield, and physical cured leaf quality. Also included in the field evaluation were non-transformed K326 (certified seed), ‘NC95, ’ and ‘LAFC53.’ LAFC53 is a low alkaloid, *nic1nic1 nic2nic2* nearly isogenic version of flue-cured tobacco cultivar NC 95 that was chosen for comparison with the RNAi DH lines that were also expected to exhibit a low alkaloid phenotype.

The thirteen selected lines were evaluated in field experiments carried out during 2012 at the Oxford Tobacco Research Station (Oxford, NC) and the Upper Coastal Plain Research Station (Rocky Mount, NC). The experimental design in each environment was a randomized complete block design with four replications. Plots consisted of single 20-plant rows and were managed according to standard flue-cured production practices for North Carolina. Intra-row and inter-row plant spacing was 56 cm and 122 cm, respectively, at both locations.

Leaves were harvested in four separate harvests (primings) and flue-cured. Each priming was weighed to generate yield data, and official USDA quality grades were assigned by a former USDA grader. A numerical reflection of physical cured leaf quality for each plot was generated for the Oxford location using the 2012 North Carolina Flue-Cured Tobacco Grade Index [52]. Physical quality data was not generated for the Rocky Mount location. Value per hundred weight ($ cwt^-1^) for the Oxford location was calculated based on average prices paid for standard grades during the 2013 growing season. Plot values for grade index and $ cwt^-1^ were calculated using a weighted average over all four primings. Fifty-gram cured leaf samples were prepared for each plot by compositing cured leaf from each priming on a weighted-mean basis. Oven-dried samples were ground to pass through a 1-mm sieve and analyzed for leaf chemistry (expressed as a percentage of dry weight) as outlined below.

In addition, after the completion of the leaf harvests at the Oxford location, root samples were collected from a single plant from each plot, oven-dried, and ground to pass through 1-mm sieve. Ground root samples were analyzed for alkaloid profiles as outlined below.

### Field Evaluation of Mutation-Derived *BBL* Materials

Sexual crossing, self-pollinations, and SNP genotyping were used to assemble the truncation mutations in *BBLa*, *BBLb*, and *BBLc* into one of seven different possible homozygous genotypic classes (Fig. 2). Triple homozygous wild type segregants were also identified from this activity. Plants of each genotype were evaluated in a field experiment at the Central Crops Research Station (Clayton, NC) during the 2012 growing season. The number of plants in each genotypic class was variable and ranged from four to thirteen. Intra-row and inter-row plant spacing was 56 cm and 122 cm, respectively. The terminal inflorescence of all individuals was removed after flowering to stimulate alkaloid accumulation. Thirteen days after removal of the inflorescence, the third and fourth leaves from the top of each plant were collected, air-cured, ground to pass through a 1-mm sieve, and analyzed for leaf alkaloids as outlined below.

### Chemical Analyses

Tobacco samples were prepared by weighting 0.2000 g of ground leaf into 50 ml Erlenmeyer flasks. Two ml of a 2 N NaOH solution was added to each sample. Flasks were swirled gently to thoroughly moisten the ground samples and were allowed to sit for 15 min. Ten ml of methyl tert-butyl ether (MTBE) solution containing an internal standard consisting of 0.40 mg/ml quinolone was added to each flask. Stoppers covered in aluminum foil were place into each flask before shaking at high rpm for 2.5 h. Flasks were allowed to sit overnight to permit layer separation, and aliquots of the MTBE layer were subjected to gas chromatographic analysis.

Quantitative determinations of nicotine, nornicotine, anabasine, anatabine, and DMN in ground leaf and root samples were made via gas chromatographic analyses using an Agilent HP 6890 GC-FID and a 30 m DB-5MS column (J & W 0.53 mm i.d and 1.50 μm film thickness) with a 1 m deactivated guard column in front. The carrier gas was Helium at a linear gas velocity of approximately 38 cm/s. The injector was set at 250°C and the detector at 325°C. The analysis consisted of a temperature program from 110°C held for 1 minute to 200°C at 10°C/min followed by an increase to 300°C at 25°C/min. Temperature was held at 300°C for 10 min. Identity of compounds of interest was verified by comparison of retention times to that of authentic standards and/or confirmed by GC-MS analysis using an HP 5890 GC equipped with an HP5972 series MSD detector. The GC-MS system was equipped with a 30 m Restek RTX-5MS column (0.25 mm i.d., 0.25 μm film thickness) and operated using the same instrument parameters used in the GC-FID analyses. The ionization voltage was 70 eV. GC-FID quantification of compounds utilized an internal standard method. Results were reported as percentages on a dry-tobacco-weight basis. Percent total alkaloids was reported as the sum of % nicotine, % nornicotine, % anabasine, % anatabine, and % DMN. Percent reducing sugars for cured leaf samples was quantified using the method of Davis [53].

### Statistical Analyses

For measured trait data generated from field evaluation of the RNAi materials and associated checks, an analysis of variance appropriate for analyzing a randomized complete block design was conducted using PROC GLM of SAS 9.3 (SAS Institute, Cary, NC). Entries and environments were considered fixed and random effects, respectively. Censored observations (those below the minimum limit of detection) were replaced by one-half the detection limit according to Atwood et al. [54]. Separation of entry means was performed using Fisher’s least significant difference test (alpha α = 0.05) [55].

For comparison of genotypic class means for mutation-derived materials, averages were first calculated for the quantified alkaloids. Genotypic class means were compared using calculated 95% confidence intervals.

## Supporting Information

S1 Fig Preparation of pKYLX71 RNAi binary vector for silencing of the *BBL* gene family. (TIF)

S1 Table In silico analysis of BBL gene expression. (XLSX)

S2 Table Oligonucleotide primers used in PCR and DNA sequencing for screening point mutations in targeted alkaloid biosynthetic genes. (XLSX)
