# Supplementary figures and images for: Transgenic and Mutation-Based Suppression of a Berberine Bridge Enzyme-Like (BBL) Gene Family Reduces Alkaloid Content in Field-Grown Tobacco
Source: PLoS One. 2015 Feb 17;10(2):e0117273. doi: 10.1371/journal.pone.0117273 (PMC4331498; doi:10.1371/journal.pone.0117273)

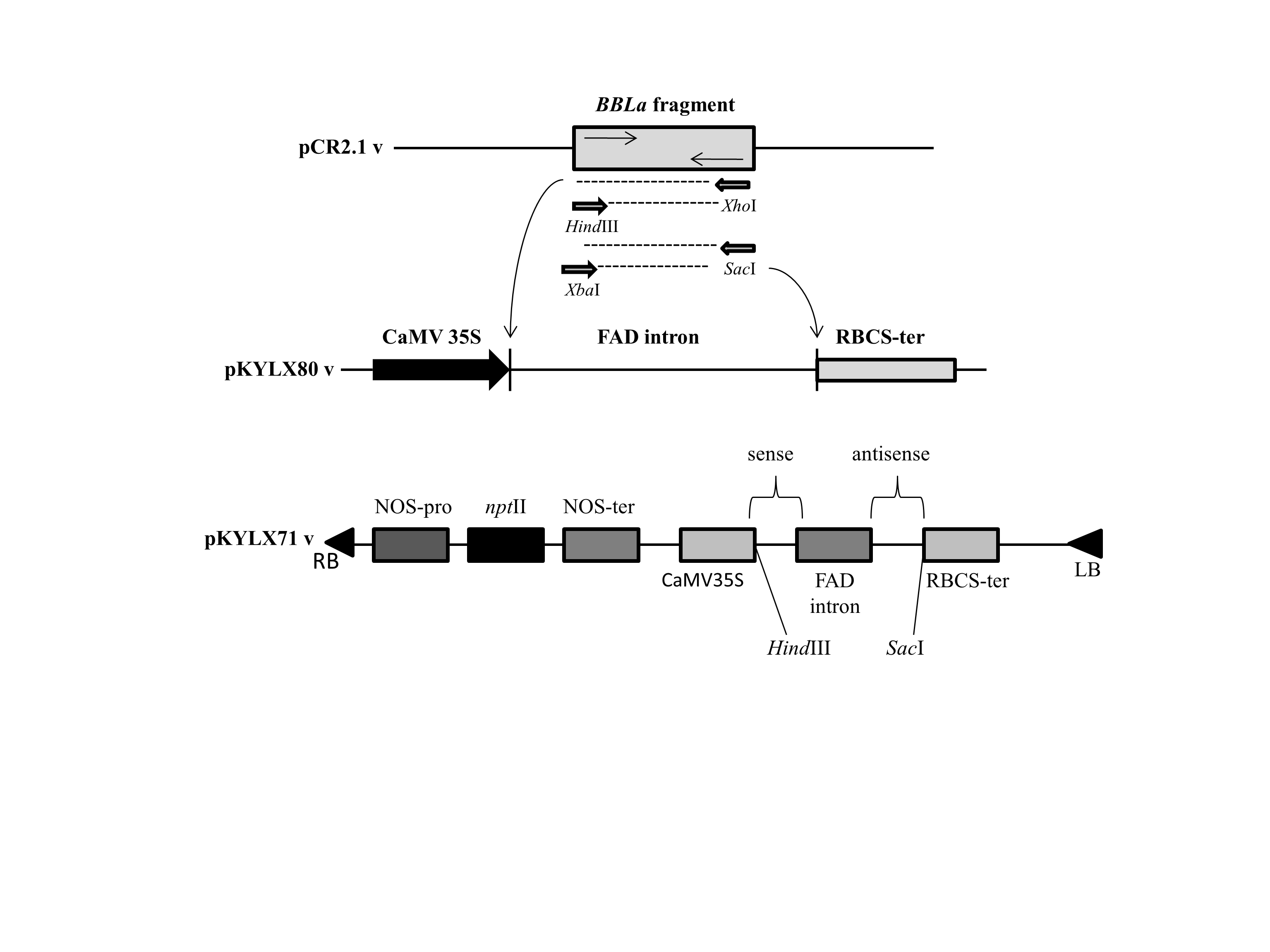

Supplement: S1 Fig — (TIF) [file pone.0117273.s001.tif]
